# The latest HyPe(r) in plant H_2_O_2_ biosensing

**DOI:** 10.1093/plphys/kiab306

**Published:** 2021-07-02

**Authors:** José Manuel Ugalde, Michelle Schlößer, Armelle Dongois, Alexandre Martinière, Andreas J. Meyer

**Affiliations:** 1 INRES—Chemical Signalling, University of Bonn, Friedrich-Ebert-Allee 144, 53113 Bonn, Germany; 2 BPMP, Univ Montpellier, CNRS, INRAE, Institut Agro, Montpellier, France

## Abstract

HyPer7 senses minute amounts of H_2_O_2_ independent of pH and the glutathione redox potential and enables detection of physiological H_2_O_2_ fluxes within the cytosol and between subcellular compartments.

Dear Editor,

Hydrogen peroxide (H_2_O_2_) is widely used as a signaling molecule in plants during development, wounding, pathogen or symbiotic interaction, and a wide range of abiotic stresses (Waszczak et al., 2018; Smirnoff and Arnaud, 2019). For better understanding its role as a messenger, it is critical to measure H_2_O_2_ with high spatial and temporal resolution (Gilroy et al., 2016). Stress-induced oxidation has frequently been shown with oxidation-sensitive fluorescent chemical probes, most prominently 2′,7′-dihydrodichlorofluorescein diacetate (Fichman et al., 2019). These probes, however, have the disadvantage of limited specificity for H_2_O_2_ and the fact that they may report only the accumulation of oxidants over time without any dynamic and spatial information (Winterbourn, 2014; Ortega-Villasante et al., 2018). In addition, these probes require loading into cells and subsequent deesterification to become active, and they can be analyzed only intensiometrically with excitation at a single wavelength. Altogether, this renders the observed fluorescence sensitive to several non-controlled factors and thus often ambiguous. Genetically encoded probes for H_2_O_2_ have the promise of overcoming these limitations (Meyer and Dick, 2010; Schwarzländer et al., 2016). Probes of the HyPer family consist of a circularly permuted yellow fluorescent protein (cpYFP) core and a sensing domain constructed from the bacterial transcription factor hydrogen peroxide-inducible genes activator (OxyR) (Belousov et al., 2006; Bilan and Belousov, 2016). cpYFP, however, has the disadvantage of a pronounced pH-sensitivity (Schwarzländer et al., 2014). With a more alkaline cytosolic pH in illuminated green tissues as measured with cpYFP, HyPer readouts of H_2_O_2_ fluxes become highly ambiguous and demand elaborate controls (Exposito-Rodriguez et al., 2017). In contrast, roGFP2-Orp1 is pH-insensitive and has successfully been used to sense H_2_O_2_ originating from an elicitor-induced oxidative burst in the apoplast and from chloroplasts in which reactive oxygen species production was boosted by methyl viologen (MV) in combination with light (Nietzel et al., 2019; Ugalde et al., 2021). Small dynamic changes in H_2_O_2_ in the low nanomolar range may, however, not be easily detectable because of a limited responsiveness of Orp1 to H_2_O_2_ and it may be overridden by a strong reducing effect of glutathione. Indeed, mutants with a less negative glutathione redox potential (*E*_GSH_) render the sensor more responsive due to the diminished reducing power (Marty et al., 2009; Nietzel et al., 2019).

Recently, an ultrasensitive HyPer variant, HyPer7, consisting of the OxyR protein of *Neisseria meningitidis* and a mutated cpYFP that is largely pH-insensitive, was reported (Pak et al., 2020). These features sparked much interest across biology and thus we are currently observing multiple laboratories considering the use of HyPer7. To test its ability for reporting dynamic changes of H_2_O_2_ in the cytosol of Arabidopsis (*Arabidopsis thaliana*), we generated stable HyPer7 reporter lines. These plants did not show any obvious phenotype (Supplemental Materials and Methods and Supplemental Figure S1). Ratiometric analysis of HyPer7 indicated that the sensor was largely reduced in all tissues (Supplemental Figure S1). Depletion of glutathione by germinating seeds on l-buthionine sulfoximine (BSO) (Meyer et al., 2007) caused only a slight ratio increase in HyPer7 while the original HyPer (Costa et al., 2010) was unresponsive and roGFP2-Orp1 (Nietzel et al., 2019) was fully oxidized (Supplemental Figure S2). This suggests that reduction of HyPer7 is largely independent of *E*_GSH_, which is consistent with recent findings in yeast (Kritsiligkou et al., 2021). With GSH depletion, H_2_O_2_ concentrations increase to levels that can be sensed by HyPer7. To further elucidate the responsiveness of HyPer7 to an externally imposed oxidation, we perfused seedlings with H_2_O_2_, buffer, and dithiothreitol (DTT) and compared its response with reporter lines expressing roGFP2-Orp1 and HyPer (Supplemental Materials and Methods and Figure 1, A). When perfused with H_2_O_2_, all three sensors showed a concentration-dependent increase of the fluorescence ratio with HyPer7 showing the largest fold-change of about 4 compared with 3.2 for roGFP2-Orp1 and less than 2 for HyPer with 1 mM H_2_O_2_ perfusion (Figure 1, B–E). The speed of oxidation of HyPer7 after perfusion with H_2_O_2_ was slightly faster than for the other sensors. Cytosolic changes in H_2_O_2_ concentrations after perfusion with 0.01 mM H_2_O_2_ were only visible with HyPer7 (Figure 1, F–H). Subsequent perfusion with buffer consistently led to a decline in the fluorescence ratio. Addition of 10 mM DTT 6 min after the washout of H_2_O_2_ caused a further drop in ratio back to the starting value for roGFP2-Orp1 consistent with the response of other roGFP2-based probes (Marty et al., 2009), but did not cause any distinct change for HyPer7 (Figure 1, B–E and Supplemental Figure S3, A). This suggests that DTT is not capable of efficiently reducing the OxyR domain. Consistent with this observation, 10 mM DTT exerted only a minor reducing effect on recombinant HyPer7 while 5 mM of the stronger reductant Tris(2-carboxyethyl)phosphine hydrochloride (TCEP) reduced the protein completely (Supplemental Figure S3, B).

**Figure 1:**
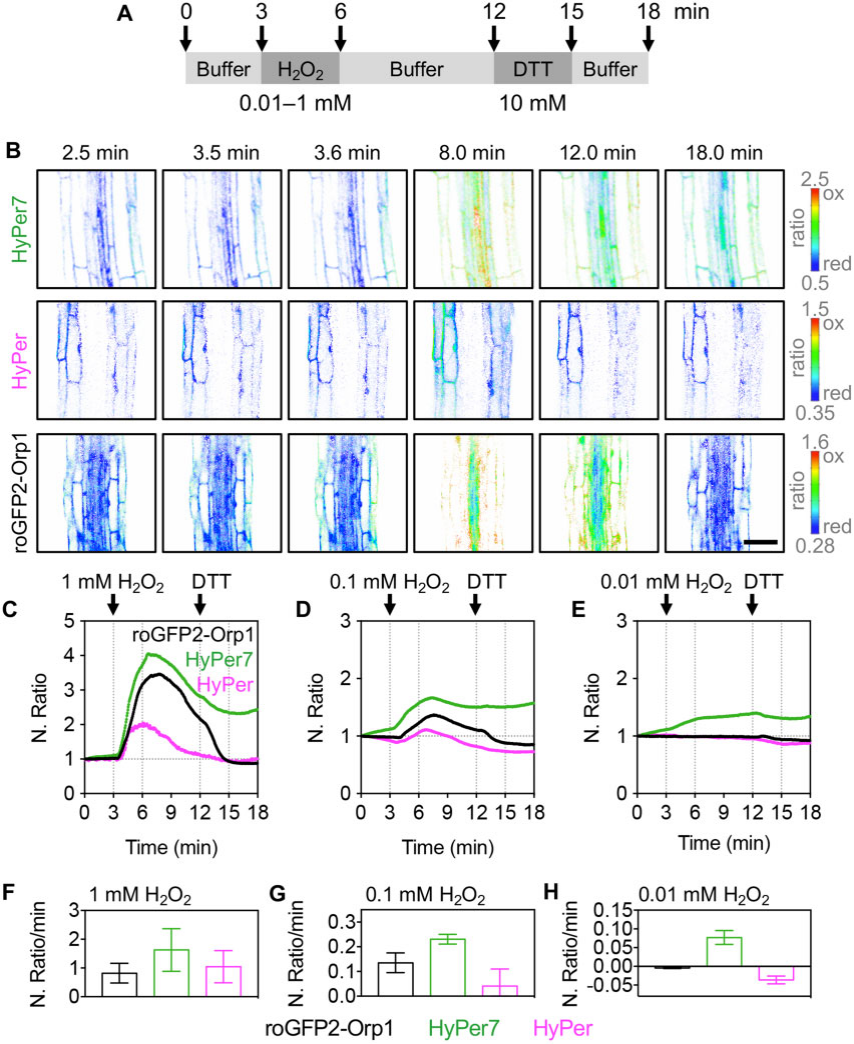
Oxidation and reduction of different genetically encoded H_2_O_2_ probes in the cytosol of wild-type Arabidopsis seedlings. A, Experimental design for sequential perfusion with different concentrations of H_2_O_2_, imaging buffer and 10 mM DTT. B, Confocal microscopy images of root cells from 7-d-old seedlings expressing HyPer7, HyPer, or roGFP2-Orp1, all targeted to the cytosol. The false color ratio images show the fluorescence ratios calculated from two separate images collected with excitation at 488 and 405 nm. Emission was collected at 508–535 nm. Ratios are 488/405 nm for HyPer7 and HyPer, and 405/488 nm for roGFP2-Orp1. Bar, 50 μm. C–E, Typical time courses showing the dynamic response of the probes to transient oxidation and subsequent reduction by the indicated treatments (arrows). All ratios are normalized to the starting values at *t* = 0 min. *n* = 2–4 replicates. F–H, Slopes of the ratio value changes after H_2_O_2_ perfusion in the indicated concentrations. The slopes were calculated from ratio values between 3.9 and 4.7 min. Data indicate the mean values ± sd. *n* = 2–4 replicates.

Direct comparison of HyPer and HyPer7 for their response to externally added H_2_O_2_ in roots consistently showed a more pronounced response of HyPer7 (Supplemental Figure S4). While HyPer showed an oxidation response to 10 mM H_2_O_2_ in leaves, the same H_2_O_2_ concentration did not cause a ratio increase in roots, or even caused a complete lack of excitability at 488 nm preventing any ratiometric analysis. A pronounced drop in the fluorescence ratio of cpYFP with H_2_O_2_ identifies the decrease of the HyPer ratio to be caused by H_2_O_2_-induced acidosis in the cytosol (Supplemental Figure S5). pH-clamp experiments on roots showed the pronounced pH-dependence of HyPer while HyPer7 is largely pH-independent at neutral and slightly alkaline pH (Supplemental Figure S6). This pH-insensitivity of HyPer7 avoids ambiguities in the interpretation of measured ratio values.

To explore the capability of HyPer7 for sensing stress-induced H_2_O_2_ in live plant cells, we tested the response to oxidative stress generated at the photosynthetic electron transport chain. In leaves, 50 µM MV in combination with light causes the formation of H_2_O_2_, which is detectable with roGFP2-based probes (Ugalde et al., 2021). The response of HyPer7 suggests that HyPer7 is more sensitive than roGFP2-Orp1 and that 50 µM MV already resulted in a pronounced cytosolic oxidation with the normal plate reader excitation light (Supplemental Figure S7, A). Continuous illumination with actinic light for 1 h caused a strong oxidation, which quickly disappeared after the illumination period. Similarly, roGFP2-Orp1 can be used to monitor the cytosolic response to an apoplastic oxidative burst elicited by flg22 (Nietzel et al., 2019). While HyPer under the conditions used was not sensitive enough to monitor this burst, the response of HyPer7 was more pronounced than that of roGFP2-Orp1 (Supplemental Figure S7, B). The response of HyPer7 also indicated an additional, yet to be characterized, earlier oxidation peak about 30 min after addition of flg22, which was not observed with roGFP2-Orp1. HyPer7 thus may allow generating information that complements previous findings.

In pavement cells of cotyledons, illumination by laser light during scanning already caused a gradual oxidation of HyPer7, which increased depending on the zoom factor (Figure 2 and Supplemental Figure S8). Surprisingly, this oxidation was almost completely abolished when seedlings were pretreated with 20 µM 3-(3,4-dichlorophenyl)-1,1-dimethylurea (DCMU) as an inhibitor of photosynthetic electron transport (Figure 2, B, D, and E). Imaging of recombinant HyPer7 with the same instrument settings did not result in altered fluorescence ratios and thus did not show any signs of a putative photoconversion (Figure 2, C). Together these results suggest that laser light used for excitation is sufficient to induce H_2_O_2_ release from chloroplasts, and that these low amounts of H_2_O_2_ can be detected by HyPer7. Laser-induced oxidation of HyPer declined within minutes indicated by a decreasing fluorescence ratio (Figure 2, F–H). The capability of sequentially repeating this oxidation–reduction cycle emphasizes the dynamic response of HyPer7 albeit with a slower reduction phase.

**Figure 2:**
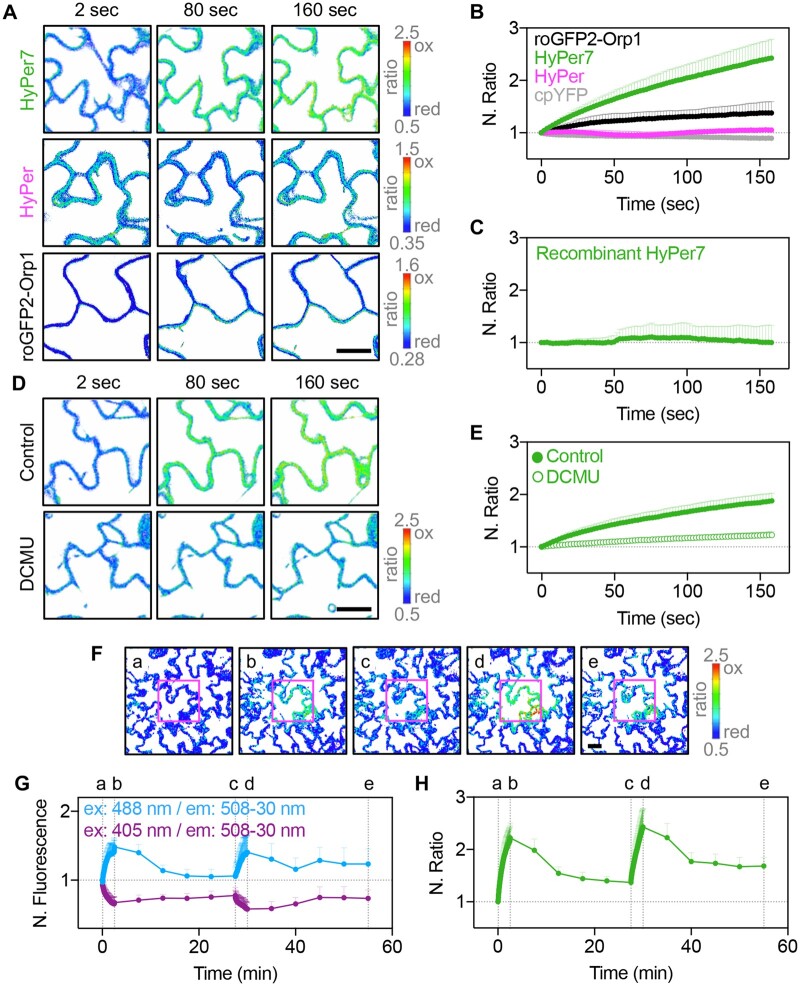
Laser illumination of green tissues during scanning is sufficient to produce release of H_2_O_2_ from chloroplasts. A and B, Laser-induced oxidation is more visible in cells expressing HyPer7 compared with cells with HyPer or roGFP2-Orp1. Plants expressing cpYFP were included to test for putative concomitant pH changes. The false color ratio images show the fluorescence ratios calculated from two separate images collected with excitation at 488 and 405 nm. Emission was collected at 508–535 nm. Ratios are 488/405 nm for HyPer7, HyPer, and cpYFP, and 405/488 nm for roGFP2-Orp1. Bar, 20 μm. C, Ratio values of purified recombinant protein imaged with the same instruments settings as in (A). The protein was reduced with TCEP prior to the measurement. D and E, Inhibition of photosynthetic electron transport blocks laser induced oxidation of HyPer7 in the cytosol. Seedlings were incubated in imaging buffer supplemented with 20 µM DCMU for 45 min in the dark prior to the measurement. Bar, 20 μm. F–H, Laser-induced oxidation of HyPer7 in a defined region of interest (magenta square) is reversible. Letters indicate time windows for laser-induced oxidation caused by high-frequency imaging at high zoom (a–b and c–d) and a recovery phase with intermittent imaging at low zoom (b–c and d–e). G, Fluorescence measured from the two independent channels (405 and 488 nm), normalized to the value at *t* = 0 min. H, 488/405 nm ratio for the values shown in panel G. All ratios in B, C, E, and H are normalized to the ratio value at *t* = 0 min. Ratios are means + sd, *n* = 4–11 replicates.

In summary, HyPer7 is largely pH-insensitive and more sensitive to H_2_O_2_ than current probes. Nonetheless, the Arabidopsis cytosol appears to lack an efficient reduction system for HyPer7, which may limit its use for fast dynamic studies. With increased sensitivity, HyPer7 offers the possibility of detecting physiological H_2_O_2_ fluxes and thus opens the door for further studies on intracellular H_2_O_2_ signaling during normal development and under environmental stress. At the same time the high sensitivity, at least in green tissues, bears the risk of generating artifacts if non-photosynthesis-related H_2_O_2_ signaling processes are to be investigated. Knowing these potential artifacts should help designing appropriate control experiments and with that generate data that help elucidate H_2_O_2_ signaling.

## Supplemental data

The following materials are available in the online version of this article.

**Supplemental Figure S1.** Expression of the HyPer7 sensor in Arabidopsis.

**Supplemental Figure S2.** Effect of GSH depletion on the oxidation of genetically encoded H_2_O_2_ probes.

**Supplemental Figure S3.** Excitation spectra of HyPer7 in vivo and in vitro.

**Supplemental Figure S4.** Determination of minimum and maximum oxidation of different H_2_O_2_ probes.

**Supplemental Figure S5.** Hydrogen peroxide causes acidification in the cytosol.

**Supplemental Figure S6.** Response of HyPer and HyPer7 to changes in intracellular pH.

**Supplemental Figure S7.** MV-induced photo-oxidative stress and the elicitor flg22 cause oxidation of H_2_O_2_ probes in the cytosol.

**Supplemental Figure S8.** Oxidation of HyPer7 depends on laser light intensity reaching the scanned area.

**Supplemental Materials and Methods.** Methodology used in this study.

## Supplementary Material

kiab306_Supplementary_Data

## Notes

A.J.M., A.M., A.D., and J.M.U. designed the experiments and analyzed the data. M.S., A.D., A.M., and J.M.U. performed the experiments. A.J.M. and A.M. supervised the experiments. A.J.M. and J.M.U. wrote the manuscript.

The author responsible for distribution of materials integral to the findings presented in this article in accordance with the policy described in the Instructions for Authors (https://academic.oup.com/plphys/pages/general-instructions) is: Andreas J. Meyer (andreas.meyer@uni-bonn.de).
