# Supplementary material for: The latest HyPe(r) in plant H2O2 biosensing
Source: Plant Physiol. 2021 Jul 2;187(2):480–4. doi: 10.1093/plphys/kiab306 (PMC8491017; doi:10.1093/plphys/kiab306)
Supplement: kiab306_Supplementary_Data [file kiab306_supplementary_data.pdf]

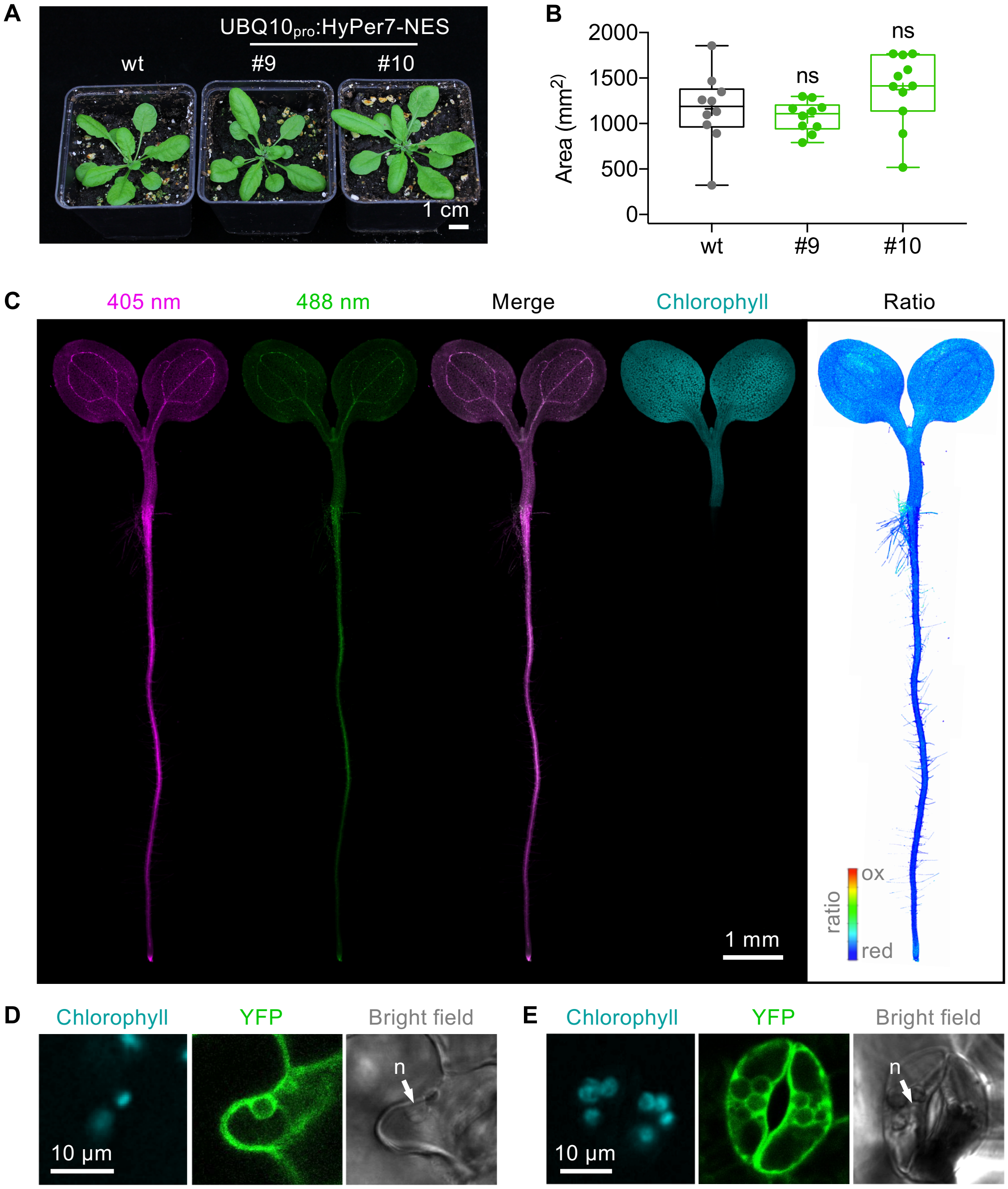

Supplemental Figure S1. Expression of the HyPer7 sensor in Arabidopsis. A, B, Stable ubiquitous expression of HyPer7-NES under control of $UBQ10_{pro}$ in the cytosol of Arabidopsis does not cause any macroscopically visible phenotypic change compared to wild-type plants. Representative image of 2 different 4-week-old lines (#9 and #10) expressing HyPer7 (A) and quantification of the rosette size (B). Box = interquartile range between the lower and upper quartiles, center line = median, + = mean, whiskers = min and max values. *n* = 10. C, Confocal microscopy images from 7-day-old seedlings expressing HyPer7. Magenta: λ_{ex} = 405 nm; λ_{em} = 508-535 nm; green: λ_{ex} = 488 nm; λ_{em} = 508-535 nm, and chlorophyll autofluorescence: λ_{ex} = 405 nm; λ_{em} = 652-679 nm). The ratio image shows the 488 nm /405 nm ratio in false color. D-E, Subcellular localization of HyPer7 in the cytosol excluding the nucleoplasm in epidermal cells (D) and guard cells (E) of 7-day-old seedlings. Arrow heads indicate the nuclei (n).

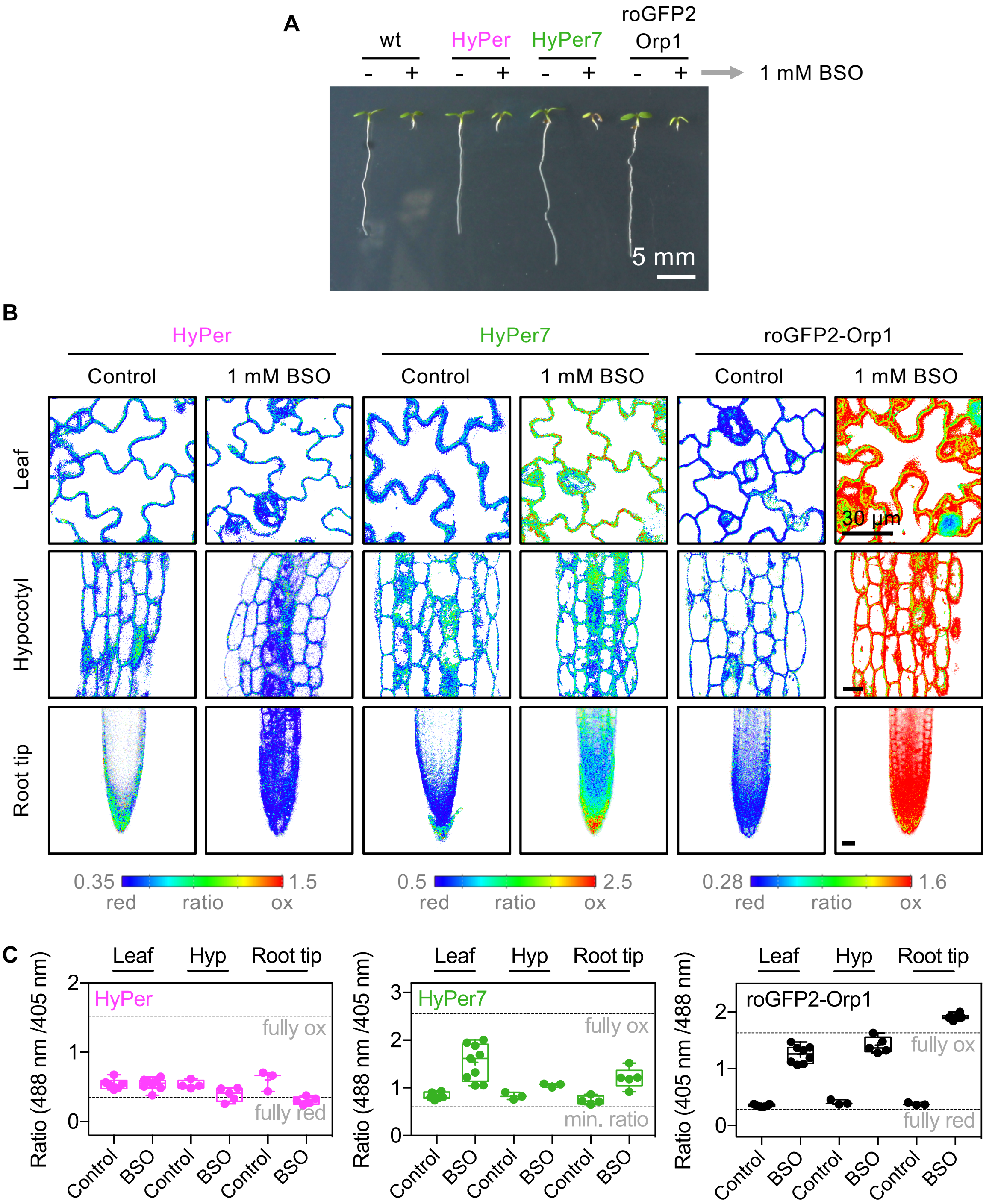

Supplemental Figure S2. Effect of GSH depletion on the oxidation of genetically encoded H_2O_2 probes. A, Phenotype of Arabidopsis seedlings stably expressing HyPer7, HyPer or roGFP2-Orp1 five days after germination on MS-plates (Control; -) or MS-plates supplemented with 1 mM L-Buthionine sulfoximine (BSO; +) as an inhibitor of glutathione biosynthesis. B, False color ratio images of leaves, hypocotyl (Hyp), and root tips from the seedlings shown in (A). Ratio images were calculated from two fluorescence channels (Ch1: λ_{ex} = 488 nm; λ_{em} = 508-535 nm and Ch2: λ_{ex} = 405 nm; λ_{em} = 508-535 nm). Ratios for HyPer and HyPer7 are calculated as 488 nm / 405 nm, and 405 nm / 488 nm for roGFP2-Orp1. C, Ratio values from leaf, root and root tip samples. Box = interquartile range between the lower and upper quartiles, center line = median, + = mean, whiskers = min and max values. *n* = 3–9. Dashed lines indicate ratios of fully oxidized sensors after treatment with 10 mM H_2O_2, or fully reduced sensors after incubation with 10 mM DTT, respectively. Because HyPer7 cannot be reduced by DTT, the lower dashed line indicates the minimum ratio measured at steady state in non-stressed seedlings.

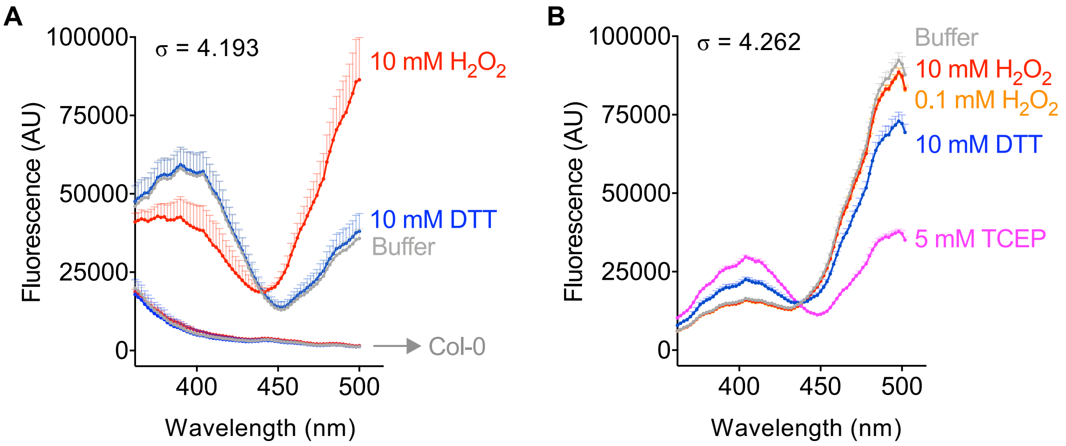

Supplemental Figure S3. Excitation spectra of HyPer7 *in vivo* and *in vitro.* A, B, Excitation spectra (λ_{ex} = 362–500 nm; λ_{em} = 520±5 nm; bandwidth = 2 nm) from 4-week-old leaf disks of plants expressing HyPer7 (A), or 1 µM purified HyPer7 sensor (B). A, Leaf disks were placed in a black 96-well plate and covered by imaging buffer (Buffer) or buffer supplemented with either 10 mM DTT or 10 mM H_2O_2 to fully reduce or oxidize the sensor, respectively. B, Purified protein was incubated in 10 mM Tris-HCl Buffer, pH = 7.4 alone (Buffer) or supplemented with either 10 mM DTT or 5 mM TCEP to reduce the sensor, or with 0.1 or 10 mM H_2O_2 to oxidize the sensor. Samples were incubated for 10 minutes before the measurement. Fluorescence was measured with a plate reader equipped with a monochromator. Mean ratios + SD, *n* = 3–6 replicates.

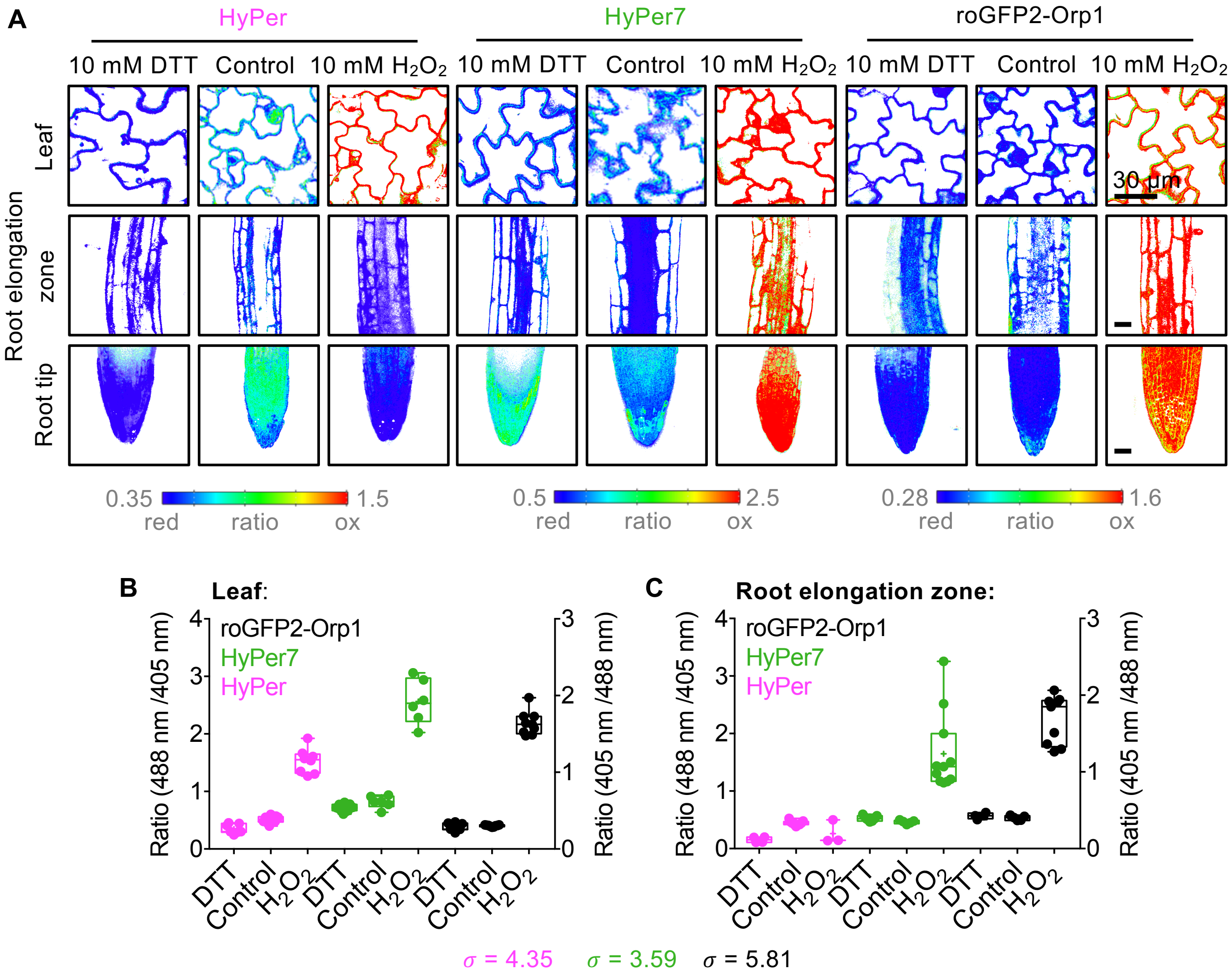

Supplemental Figure S4. Determination of minimum and maximum oxidation of different H_2O_2 probes. A, Ratio images of different tissues from 7-day-old seedlings stably expressing HyPer7, HyPer or roGFP2-Orp1 sensors in the cytosol. The false color ratio images were calculated from two fluorescence images (Ch1: λ_{ex} = 488 nm; λ_{em} = 508-535 nm and Ch2: λ_{ex} = 405 nm; λ_{em} = 508-535 nm). Ratios for HyPer and HyPer7 are calculated as 488 nm / 405 nm, and 405 nm / 488 nm for roGFP2-Orp1. Seedlings were vacuum infiltrated with either imaging buffer (Control) or buffer supplemented with 10 mM DTT or 10 mM H_2O_2 to fully reduce or oxidize the sensors, respectively. B-C, Ratio values from the leaf (B) and root elongation zone (C). Box = interquartile range between the lower and upper quartiles, center line = median, + = mean, whiskers = min and max values. σ = dynamic range of each sensor, calculated from the ratios values between fully reduced and fully oxidized probes. *n* = 6–11.

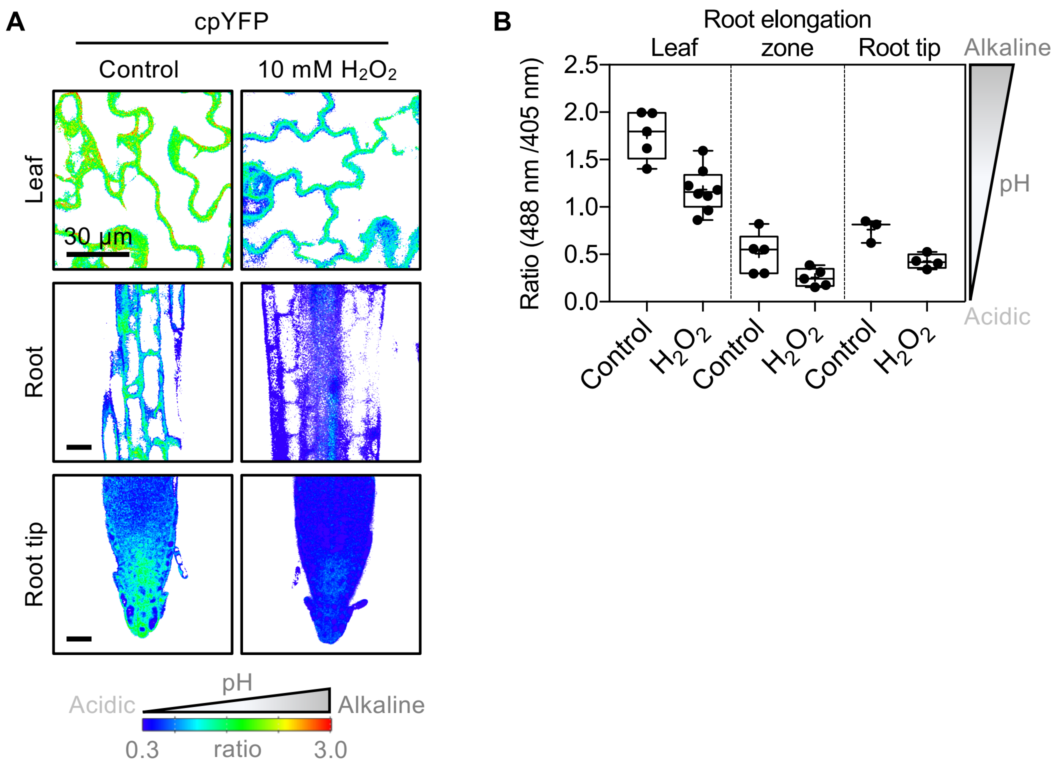

Supplemental Figure S5. Hydrogen peroxide causes acidification in the cytosol. A, Ratio images calculated from confocal images of leaf, root elongation zone and root tip of 7-day-old Arabidopsis seedlings expressing cpYFP in the cytosol. The false color 488 nm/405 nm ratio images were calculated from two individual fluorescence images (Ch1: λ_{ex} = 488 nm; λ_{em} = 508-535 nm and Ch2: λ_{ex} = 405 nm; λ_{em} = 508-535 nm). Seedlings were vacuum infiltrated with 10 mM H_2O_2 or buffer as control. B, Quantitative analysis of ratio values. Box = interquartile range between the lower and upper quartiles, center line = median, + = mean, whiskers = min and max values. *n* = 3–8.

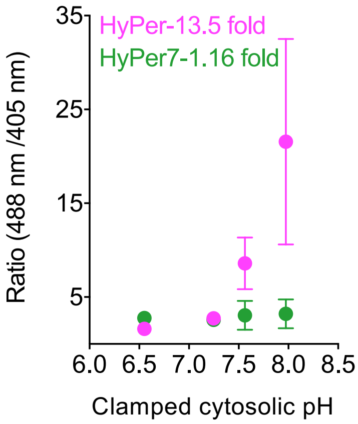

Supplemental Figure S6. Response of HyPer and HyPer7 to changes in intracellular pH. Arabidopsis seedlings with stable expression of the HyPer variants were grown on agar plates for five days and incubated for 1.5 hours in solutions containing 50 mM NaAc and buffers with different pH. The cytosolic pH was estimated by using plants expressing the pH sensor pHluorin (see Supplemental Methods). Fluorescence was recorded by confocal microscopy (Ch1: λex = 488nm; λem = 505-550 nm; Ch2: λex = 405 nm; λem = 505-550 nm). Data show mean ratio values obtained from pH-clamped root cells and error bars correspond to the 95% confidence interval. *n* = 10-12 cells from two biological replicates.

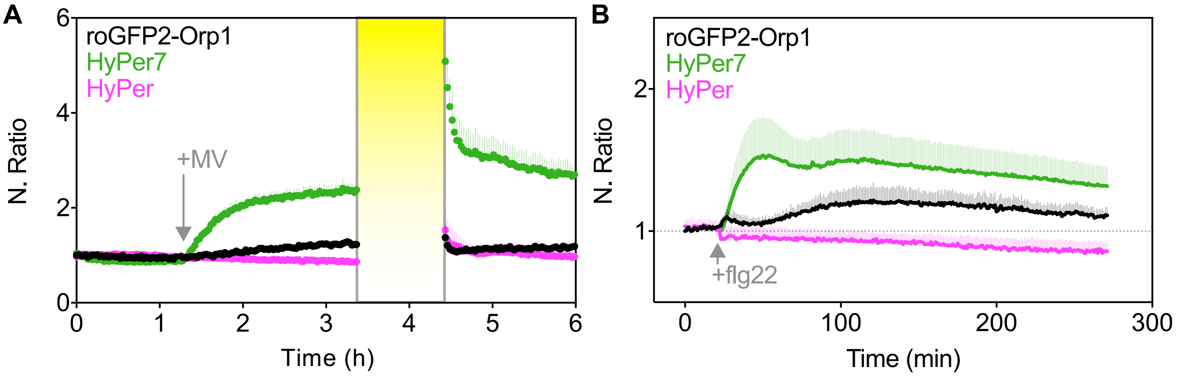

Supplemental Figure S7. Methyl viologen (MV)-induced photo-oxidative stress and the elicitor flg22 cause oxidation of H_2O_2 probes in the cytosol. A, Four-week-old leaf disks stably expressing the indicated sensors were placed in a 96-well plate with imaging buffer. After around 1 h, MV was added to a final concentration of 50 µM (indicated by the arrow). Sensor oxidation was recorded as the normalized ratio of the fluorescence in two channels (Ch1: λ_{ex} = 400±5 nm; λ_{em} = 520±5 nm and Ch2: λ_{ex} = 482±8 nm; λ_{em} = 520±5 nm). Ratios for HyPer and HyPer7 are calculated as 482 nm / 400 nm, and for roGFP2-Orp1 as 400 nm / 482 nm. After a pre-incubation with MV for 2 h, seedlings were intermittently illuminated for 1 h with actinic light (200 µmol m^{-2} s^{-1}) and measurements were subsequently resumed for additional 2 hours. Data represent mean ratios + SD, *n* = 3–4. B, Leaf disks from six-week-old plants stably expressing the indicated sensors were placed in a 96-well plate in imaging buffer. The arrow indicates addition of flg22 to a final concentration of 10 µM. Sensor oxidation was recorded as in (A). Data represent mean ratios + SD, *n* = 4–8. All ratio values were normalized to the ratio at t = 0 min.

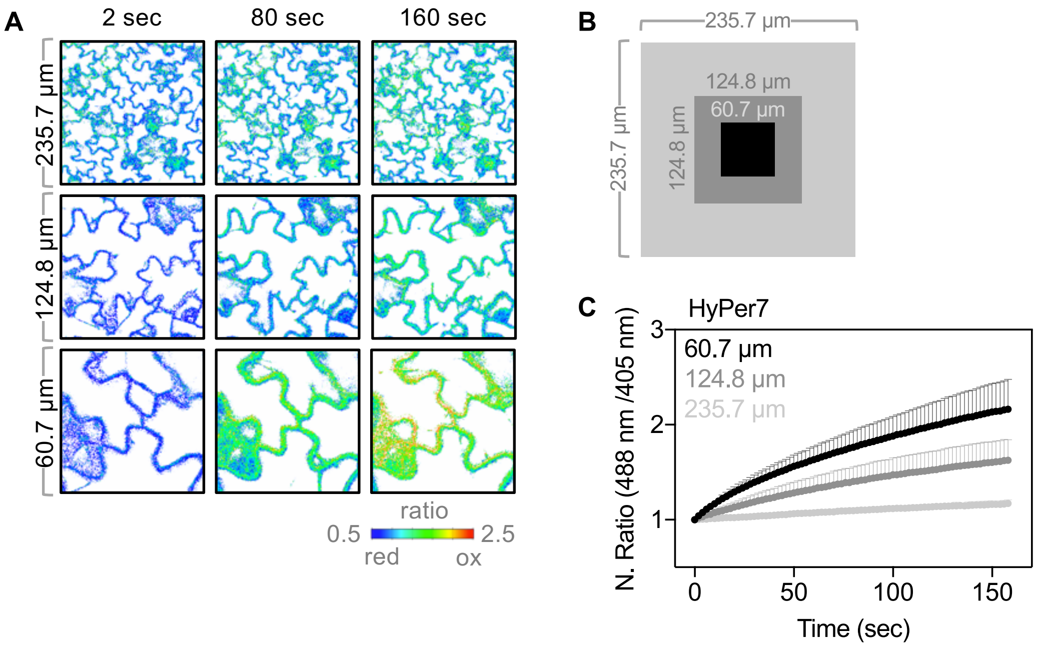

Supplemental Figure S8. Oxidation of HyPer7 depends on laser light intensity reaching the scanned area. A, Ratio images of cytosolic HyPer7 in leaf epidermal cells from 7-day-old seedlings. False color images show the 488 nm/405 nm ratio from two individual channels (Ch1: λ_{ex} = 488 nm; λ_{em} = 508-535 nm and Ch2: λ_{ex} = 405 nm; λ_{em} = 508-535 nm) recorded with different zoom factors. B, Scheme showing the different scan areas. C, Time courses for the gradual laser-induced oxidation of HyPer7 observed in the indicated scan areas. Ratio values were normalized to the values recorded at t = 0 min. Mean ratios + SD, *n* = 12–7 replicates.

MATERIAL AND METHODS

Plant material and growth conditions

Arabidopsis (*Arabidopsis thaliana*) Col-0 ([L.] Heynh.) seeds were obtained from NASC (www.arabidopsis.info). Transgenic Col-0 lines expressing HyPer and roGFP2-Orp1 targeted to the cytosol has been described earlier by (Costa et al., 2010; Nietzel et al., 2019). For experiments with seedlings, seeds were surface-sterilized with 70% (v/v) ethanol, rinsed three times with sterile deionized water and stratified for 48 h at 4ºC, and subsequently sown on plates with 0.5x Murashige and Skoog (MS) growth medium (Murashige and Skoog, 1962) (Duchefa Biochemie, Haarlem, The Netherlands) supplemented with 0.1% (w/v) sucrose, 0.05% (w/v) MES (pH 5.8, KOH) and 0.8% (w/v) agar. To grow seedlings devoid of glutathione, seeds were germinated on plates supplemented with 1 mM L-buthionine sulfoximine. For experiments with leaf discs, 5-day-old seedlings were transferred to soil and grown under the same controlled conditions for 4–5 weeks. Leaves were excised using a 7 mm-diameter cork borer. Rosette size was measured on 4-week-old plants using the Leaflab MATLAB script.

For pH-clamp experiments and salt treatments, plants expressing either HyPer7, HyPer (Costa et al., 2010) or pHluorin (Martinière et al., 2013) were grown on media containing 0.5x MS, 1% (w/v) sucrose, 0.8% (w/v) Agar type E and 2.5 mM MES-KOH pH 5.7. Plates were placed vertically for 5 days, with 16:8-h light dark cycles at 21°C with 70% relative humidity and a light intensity of 200 µmol m^{-2} s^{-1}.

Cloning of HyPer7 into a plant expression vector

The HyPer7 sequence including the original nuclear exclusion sequence (NES) was amplified by PCR from pCS2-HyPer7-NES (Pak et al., 2020) using the primers HyPer7_KpnI fw: AGGTACCATGCACCTGG and HyPer7_SalI rv TATGTCGACTTACAGGGTCAGC, which added restriction sites for *Kpn*I and *Sal*I. The fragment was sub-cloned into pJET1.2 (Thermo Fisher Scientific, Waltham, WA), digested with *Kpn*I and *Sal*I (Thermo Fischer Scientific) and purified from an agarose gel with the NucleoSpin® gel and PCR clean-up kit (Macherey-Nagel, Düren, Germany). For constitutive expression in plants, the fragment was ligated into the vector pBinCM, which is a modified version of the original pBinAR with a $UBQ10_{pro}$ instead of $35S_{pro}$ (Aller et al., 2013). Plasmid were confirmed by sequencing, transformed into *Agrobacterium tumefaciens* (AGL1) and used for transformation of Arabidopsis plants by floral dip (Clough and Bent, 1998).

Cloning of HyPer7 into a bacterial expression vector and protein purification

The HyPer7 sequence was amplified by PCR from pCS2-HyPer7-NES (Pak et al., 2020) using the primers HyPer7_gw f: GGGGACAAGTTTGTACAAAAAAGCAGGCTTTCATGCACCTGGCTAATGAGGA and HyPer7_gw_r:GGGGACCACTTTGTACAAGAAAGCTGGGTCCAGGGTCAGCCGCTCCAGGG, which added attB sites for Gateway cloning. The fragment was sub-cloned into pDONR201 and then

into the destination vector pETG-10a by recombination using the respective enzymes (Invitrogen, Carlsbad, CA). *Escherichia coli* (Rosetta strain) cells harboring the pETG-HyPer7-His vector were cultured in liquid LB media supplemented with 100 µg mL^{-1} ampicillin at 37ºC. Isopropyl-β-D-Thiogalactopyranoside (IPTG) at a final concentration of 1 mM was used for expression induction. Cell lysis was performed as described in (Nietzel et al., 2019) while protein purification was performed as described in (Ugalde et al., 2021).

Confocal laser scanning microscopy and image analysis

Plants and recombinant HyPer7 were imaged in a confocal laser scanning microscope (Zeiss LSM 780, connected to an Axio Observer.Z1; Carl Zeiss Microscopy, Jena, Germany) using a 5x lens (EC Plan-Neofluar 5x/0.16) or a 40x lens (C-Apochromat 40x/1.2 W Korr). Samples were mounted in imaging buffer (10 mM MES, 10 mM $MgCl_2$, 10 mM $CaCl_2$, 5 mM KCl, pH 5.8). All sensors were sequentially excited at 488 nm and 405 nm, and the fluorescence emission was collected at 508–535 nm. The laser power was adjusted according to the respective relative excitation efficiencies on the two excitation peaks of the respective probes. Imaging was performed without averaging with a pixel dwell time of 1.58 µs/pixel. Samples were scanned in areas set to 235.7 µm x 235.7 µm, 124.8 µm x 124.8 µm or 60.7 µm x 60.7 µm. Hydrogen peroxide (H_2O_2) and dithiothreitol (DTT) perfusion experiments were performed as described in (Ugalde et al., 2020) using a RC-22 perfusion chamber mounted on a P1 platform (Warner Instruments, Hamden, CT). To inhibit photosystem II (PSII), samples were pre-treated for 45 min with 20 µM 3-(3,4-dichlorophenyl)-1,1-dimethylurea (DCMU) in the dark (Sigma-Aldrich, Steinheim, Germany) dissolved in ethanol. The fluorescence ratio was calculated as 405 nm / 488 nm for roGFP2-Orp1 and 488 nm / 405 nm for HyPer and HyPer7 and normalized to the ratio values at the start of the experiments (t = 0 min). Ratiometric imaging and analysis of single plane images was performed with a custom-written MATLAB script (Fricker, 2016) or FIJI (Schindelin et al., 2012).

Multiwell plate reader measurements

Leaf disks from 4 or 6-week-old plants expressing HyPer7 or 1 µM recombinant HyPer7 were placed in black 96-well plates (Thermo Fischer Scientific). Leaf disks were submerged in 200 µL imaging buffer and the recombinant sensor was diluted in 10 mM Tris-HCl buffer (pH = 7.4). Samples were excited from 362 to 500 nm with a step width of 1 nm and the emission collected at 520±5 nm using a CLARIOstar plate reader (BMG Labtech, Ortenberg, Germany). Complete sensor reduction or oxidation in leaf disks was achieved by supplementing the imaging buffer with 10 mM DTT or 10 mM H_2O_2, respectively. Non-transformed leaf discs were treated under the same conditions and used for autofluorescence subtraction. For recombinant HyPer7, complete sensor oxidation was reached supplementing the Tris-HCl buffer with 0.1 or 10 mM H_2O_2. To reduce the sensor, 10 mM DTT or 5 mM Tris(2-carboxyethyl)phosphine hydrochloride (TCEP) (Bond-Breaker Solution™, Thermo Fischer Scientific) were added to the buffer. Time-resolved ratiometric measurements were

performed as described in (Ugalde et al., 2021). All sensors were sequentially excited with the filters 400 ± 5 nm and 482 ± 8 nm, and the fluorescence was collected at 520 ± 5 nm. Gains were adjusted to $1900_{(400\ nm)}$ and $1200_{(482\ nm)}$ for roGFP2-Orp1 and $1800_{(400\ nm,\ 482\ nm)}$ for HyPer and HyPer7. The fluorescence ratio was calculated as 400 nm / 482 nm for roGFP2-Orp1 and 482 nm / 400 nm for HyPer and HyPer7. The calculated ratios were normalized to the value at t = 0 h. To induce oxidative stress by endogenous ROS production, either methyl viologen (MV) (Sigma-Aldrich) was added at a final concentration of 50 µM, or the elicitor flg22 (Eurogentec, Seraing, Belgium) was added to a final concentration of 10 µM. After 2 h of incubation in MV, the measurement was paused and samples were exposed to actinic light with a photon flux density of 200 µmol m^{-2} s^{-1} for 1 h, after which the fluorescence recordings were resumed. For elicitor-induced ROS production, flg22 was added 20 minutes after start of recording of steady state ratio values.

pH-clamp experiments

To clamp the pH in plants, seedlings were incubated for 1.5 hours in 50 mM NaAc and either 50 mM MES-KOH pH 6.35, 50 mM MOPS-KOH pH 7.35, 50 mM TRICINE-KOH pH 8.35 or 50 mM TRICINE-KOH pH 9.35. Fluorescence measurements were carried on a Leica SP8 inverted laser scanning microscope, with a 40x water objective (HCX Plan Apochromat CS 1.1 NA) and with 2x zoom (273 nm/pixel). For each observation, two images were taken with sequential excitation at 405 nm and 488 nm, corresponding to the two excitation peaks of the fluorescent proteins. Emitted light was collected between 505 and 550 nm.

To estimate cytoplasmic pH in planta after incubation with pH-clamping solution, we used plants expressing the ratiometric pH sensor pHluorin (Martinière et al., 2013). After incubation with the different solutions, ratio values (Ch1/Ch2) were calculated from the two channels (Ch1: λex = 405 nm; λem = 505-550 nm; Ch2: λex = 488 nm; λem = 505-550 nm). Subsequently, ratios were converted to pH units based on a calibration curve made for recombinant pHluorin (Martinière et al., 2013 and 2018).

References

Aller I, Rouhier N, Meyer AJ (2013) Development of roGFP2-derived redox probes for measurement of the glutathione redox potential in the cytosol of severely glutathione-deficient *rml1* seedlings. Front Plant Sci. doi: 10.3389/fpls.2013.00506

Clough SJ, Bent AF (1998) Floral dip: a simplified method for *Agrobacterium* -mediated transformation of *Arabidopsis thaliana*. Plant J **16**: 735–743

Costa A, Drago I, Behera S, Zottini M, Pizzo P, Schroeder JI, Pozzan T, Schiavo FL (2010) H_2O_2 in plant peroxisomes: an *in vivo* analysis uncovers a Ca^{2+}-dependent scavenging system. Plant J **62**: 760–772

Fricker MD (2016) Quantitative redox imaging software. Antioxid Redox Signal **24**: 752–762

Martinière A, Bassil E, Jublanc E, Alcon C, Reguera M, Sentenac H, Blumwald E, Paris N (2013) *In vivo* intracellular pH measurements in tobacco and Arabidopsis reveal an unexpected pH gradient in the endomembrane system. Plant Cell **25**: 4028–4043

Martinière A, Gibrat R, Sentenac H, Dumont X, Gaillard I, Paris N (2018) Uncovering pH at both sides of the root plasma membrane interface using noninvasive imaging. PNAS **115**: 6488–6493

Murashige T, Skoog F (1962) A Revised Medium for Rapid Growth and Bio Assays with Tobacco Tissue Cultures. Physiol Plantarum **15**: 473–497

Nietzel T, Elsässer M, Ruberti C, Steinbeck J, Ugalde JM, Fuchs P, Wagner S, Ostermann L, Moseler A, Lemke P, et al (2019) The fluorescent protein sensor roGFP2-Orp1 monitors *in vivo* H_2O_2 and thiol redox integration and elucidates intracellular H_2O_2 dynamics during elicitor-induced oxidative burst in *Arabidopsis*. New Phytol **221**: 1649–1664

Pak VV, Ezeriņa D, Lyublinskaya OG, Pedre B, Tyurin-Kuzmin PA, Mishina NM, Thauvin M, Young D, Wahni K, Martínez Gache SA, et al (2020) Ultrasensitive genetically encoded indicator for hydrogen peroxide identifies roles for the oxidant in cell migration and mitochondrial function. Cell Metabolism **31**: 642-653.e6

Schindelin J, Arganda-Carreras I, Frise E, Kaynig V, Longair M, Pietzsch T, Preibisch S, Rueden C, Saalfeld S, Schmid B, et al (2012) Fiji: an open-source platform for biological-image analysis. Nat Methods **9**: 676–682

Ugalde JM, Fecker L, Schwarzländer M, Müller-Schüssele SJ, Meyer AJ (2020) Live monitoring of ROS-induced cytosolic redox changes with roGFP2-based sensors in plants. bioRxiv 2020.12.21.423768

Ugalde JM, Fuchs P, Nietzel T, Cutolo EA, Homagk M, Vothknecht UC, Holuigue L, Schwarzländer M, Müller-Schüssele SJ, Meyer AJ (2021) Chloroplast-derived photo-oxidative stress causes changes in H_2O_2 and E_{GSH} in other subcellular compartments. Plant Physiol. **186**: 125-141
